# A Transformer-Based Variational Autoencoder for Training Data Generation in Spindle Motor Vibration-Based Anomaly Detection

**DOI:** 10.3390/s26072176

**Published:** 2026-03-31

**Authors:** Jaeyoung Kim, Youngbae Hwang

**Affiliations:** 1 Department of Industrial Artificial Intelligence, Chungbuk National University, Cheongju-si 28644, Republic of Korea; jykim@cbnu.ac.kr; 2 Department of Intelligent Systems and Robotics, Chungbuk National University, Cheongju-si 28644, Republic of Korea

**Keywords:** Transformer-based Variational Autoencoder, vibration, spindle motor, anomaly detection, data augmentation

## Abstract

In high-speed spindle motors operating above 10,000 rpm, vibration analysis is essential for detecting mechanical anomalies. However, data scarcity and imbalance, especially for rare fault conditions, limit the performance of deep learning-based anomaly detection models. In this study, we define sample scarcity as the limited availability of real labeled vibration sequences for model training, i.e., only 5000 normal and 5000 faulty samples collected from three spindle motors (10,000 real samples in total). We propose a Transformer-based Variational Autoencoder (T-VAE) to generate realistic triaxial acceleration sequences for spindle motor health monitoring. The model integrates positional encoding and multi-head self-attention to capture long-range temporal dependencies in multivariate time-series data, and applies a KL annealing strategy to improve training stability. Using 5000 normal and 5000 faulty vibration samples collected from three spindle motors, the model generates 100,000 synthetic samples per class, which are used to augment training for a downstream CNN–LSTM classifier. Without augmentation, the classifier achieved 95.73% pass detection on normal samples and 81.40% fail detection on faulty samples. After augmentation with Transformer-VAE, performance increased to 98.07% pass detection for normal data and 97.99% fail detection for faulty data. For prediction, we evaluate on an independent dataset of 25,000 normal and 25,000 faulty sequences obtained from eleven different spindle motors not used in training (cross-spindle). The results demonstrate that the T-VAE effectively alleviates the data scarcity problem and significantly improves anomaly detection accuracy for high-speed spindle motor vibration signals. This approach can be directly applied to predictive maintenance systems in real-world manufacturing environments.

## 1. Introduction

High-speed spindle motors are essential components in modern manufacturing systems such as CNC machining centers, precision grinders, and semiconductor wafer dicing machines. These motors frequently operate above 10,000 rpm, where dynamic stability and bearing integrity directly influence machining precision, surface quality, and productivity [1,2]. Mechanical degradations—such as bearing defects, unbalance, shaft misalignment, and lubrication failures—can propagate rapidly under such high speeds, leading to catastrophic breakdowns and significant economic loss [3,4]. Consequently, vibration-based condition monitoring has become a core element of predictive maintenance frameworks across aerospace, automotive, and industrial manufacturing sectors [5].

Traditional vibration analysis relied heavily on time- and frequency-domain handcrafted features such as root-mean-square (RMS), kurtosis, crest factor, and spectral energy ratios, often followed by shallow classifiers (SVM, kNN, ANN) [3,6]. However, these approaches are limited by their dependence on domain expertise and sensitivity to noise and operating variations [6]. Recent advances in deep learning have enabled automatic extraction of hierarchical representations directly from raw vibration data, providing superior fault recognition accuracy and robustness [7]. Hybrid architectures combining convolutional and recurrent layers (CNN–LSTM) have been particularly successful for capturing both spatial and temporal dependencies in rotating machinery signals [8]. Nevertheless, the performance of such supervised learning models still relies critically on large, balanced datasets—an unrealistic assumption in industrial settings where fault data are scarce and normal operating samples dominate. This inherent data scarcity and imbalance problem remains one of the major challenges in applying deep models for real-world spindle fault diagnosis.

Beyond the general trend of deep learning-based condition monitoring, substantial progress has also been reported by European researchers and consortia in vibration-based fault diagnosis and prognostics. In particular, European benchmark efforts have emphasized realistic operating variability and repeatable evaluation protocols for data-driven diagnostics, exemplified by the Paderborn bearing dataset based on motor-current and vibration measurements [9]. These works have contributed to practical understanding of how frequency-domain fault signatures (e.g., harmonics/sidebands and low-frequency energy elevation) manifest under diverse operating conditions, complementing classical vibration-diagnostic foundations [3,4].

In industrial spindle monitoring, however, the dominant practical bottleneck is not model choice but the lack of sufficiently diverse labeled fault data and the presence of strong domain shift across different spindle units and operating regimes. Even when deep models such as CNN–LSTM achieve high in-distribution accuracy, they can degrade markedly on unseen machines unless cross-spindle separation and leakage-free evaluation are enforced [10,11,12]. Accordingly, the present work distinguishes (i) prior knowledge on vibration-feature learning and fault diagnosis from (ii) practice-driven constraints in real spindles (small-sample training with limited spindle-level diversity and strict cross-spindle generalization), which motivates generative augmentation in a Transformer-VAE framework.

To overcome this limitation, data augmentation techniques based on generative models have gained increasing attention. Generative Adversarial Networks (GANs) have demonstrated potential for producing synthetic fault-like signals to balance datasets and enhance classifier generalization [13,14]. However, GAN training often suffers from instability and mode collapse, producing limited diversity and poor physical interpretability [15]. Alternatively, Variational Autoencoders (VAEs) offer a probabilistic framework that can learn continuous latent manifolds of vibration dynamics and generate realistic reconstructions [16,17]. Several recent studies have explored conditional or hybrid variants, such as conditional-VAE (CVAE) and β-VAE, to improve controllability and disentanglement of latent features [18,19]. Despite these improvements, CNN- or RNN-based VAEs remain insufficient to capture the long-range temporal dependencies intrinsic to spindle vibration sequences under variable speeds and load conditions.

Recently, Transformer architectures—initially proposed for natural language processing—have revolutionized time-series modeling through multi-head self-attention mechanisms capable of learning global temporal dependencies [20]. In machinery fault diagnosis, Transformers have been successfully adapted for feature extraction and cross-domain generalization, outperforming conventional CNNs and LSTMs in both accuracy and interpretability [21,22,23,24]. Nevertheless, their application as generative models for vibration-based data synthesis remains largely unexplored. Moreover, ensuring that generated signals maintain not only time-domain waveform realism but also frequency-domain physical plausibility is essential for practical deployment in predictive maintenance pipelines [25].

Although several open-source datasets, such as the Case Western Reserve University (CWRU) bearing dataset [26] and the Paderborn current-signal benchmark [9], are widely used for fault diagnosis research, they mostly contain simplified laboratory setups with limited spindle-level variability. Consequently, models trained solely on such datasets often fail to generalize to real industrial conditions. To address this limitation, the present study focuses on cross-spindle data collected from high-speed industrial spindle motors under realistic operating environments. While CWRU and Paderborn remain valuable for benchmarking, they do not capture the cross-spindle domain shift targeted here; accordingly, we prioritize industrial spindles and enforce strict cross-spindle splits to evaluate generalization to unseen machines.

In this paper, we propose a Transformer-based Variational Autoencoder (T-VAE) framework to generate high-fidelity triaxial acceleration sequences for high-speed spindle motors. The proposed model integrates positional encoding and multi-head self-attention to capture long-range vibration dependencies while employing a KL annealing strategy to stabilize latent regularization and prevent posterior collapse. Using 5000 normal and 5000 faulty samples from three spindle motors, the T-VAE generates 100,000 synthetic sequences per class. These augmented datasets are then used to retrain a downstream CNN–LSTM classifier, significantly enhancing fault detection robustness. For evaluation, we conduct cross-spindle validation on eleven unseen spindle motors (25,000 normal and 25,000 faulty samples). The results show substantial improvements: baseline classification yields 95.73% accuracy for normal detection and 81.40% for fault detection, whereas T-VAE augmentation increases performance to 98.07% and 97.99%, respectively.

The main contributions of this study are summarized as follows:

- We propose a T-VAE architecture tailored for triaxial vibration data, combining self-attention with positional encoding to capture long-term mechanical dependencies.
- A KL annealing mechanism is introduced to mitigate posterior collapse and enhance latent stability.
- We empirically demonstrate that T-VAE-based augmentation improves CNN–LSTM classifier accuracy by up to 17 percentage points under cross-spindle evaluation.
- Both time- and frequency-domain analyses (FFT, PCA) show that generated signals maintain physical plausibility and vibration spectrum fidelity.
- The proposed approach effectively mitigates the data imbalance problem and provides a scalable foundation for intelligent predictive maintenance of high-speed spindles [12,27].

## 2. Related Works

### 2.1. Deep Learning for Vibration-Based Fault Diagnosis

Traditional approaches to vibration-based fault detection relied heavily on handcrafted statistical and spectral features. With the advent of deep learning, models such as CNNs and RNNs demonstrated the ability to automatically extract discriminative features from raw time-series signals [6,7]. Hybrid models, including CNN–LSTM, further improved temporal feature learning by combining spatial convolution with sequential dependencies. However, these methods remain highly data-dependent, and their performance deteriorates under data scarcity.

### 2.2. Generative Approaches for Data Augmentation

Beyond vanilla VAEs and GANs, recent works have explored conditional VAEs and β-VAEs to improve the fidelity and controllability of synthesized vibration sequences [16]. For example, Zhao et al. proposed a conditional VAE with an adaptive focal-loss scheme to explicitly address class imbalance, reporting significant gains in downstream accuracy under data scarcity [19]. In parallel, spectrum-aware generative approaches have been introduced to preserve frequency-domain characteristics of the signals—an essential requirement for spindle vibration, where characteristic bearing-fault frequencies must remain physically consistent [25]. Taken together, these trends indicate that spectrum-aware priors and class-imbalance-aware objectives are crucial to avoid implausible generations that could mislead classifiers [16,19,25].

### 2.3. Transformers for Diagnosis and Generation

On the discriminative side, Swin-Transformers and hybrid CNN–Transformer backbones have been adopted for bearing/spindle diagnosis under varying loads and speeds, showing robust feature extraction and better long-range temporal modeling than RNNs or CNNs alone [21,22,23]. For cross-domain generalization, adversarial or contrastive alignment and meta-learning have been incorporated into Transformer pipelines to bridge distribution gaps among machines, sessions, and speeds [27,28]. On the generative side, Transformer-based VAEs for sequential data have also emerged, where self-attention in the encoder and decoder improves reconstruction of long-horizon dependencies and mitigates posterior collapse compared with RNN decoders [16,20,29].

### 2.4. Evaluation Protocols and Feature Foundations

To ensure fair evaluation and avoid optimistic bias due to data leakage and improper model selection, our study follows a strict cross-spindle validation protocol, using three spindles for training and eleven unseen spindles for prediction [10,11]. We report key evaluation metrics, including accuracy, precision, recall, F1-score, ROC curves, and confusion matrices, to assess both classification performance and generalization.

The feature diagnostics employed throughout the paper—namely, FFT spectra, PCA projections, axis-wise probability density visualizations (via Gaussian-kernel KDE), and correlation matrices—are standard and theoretically grounded. For spectral analysis, we estimate power spectra using FFT-based averaged periodograms following Welch’s method [30]. PCA is carried out in the conventional manner as reviewed by Jolliffe and Cadima [31]. For density estimation, we adopt Gaussian-kernel KDE [32] with bandwidths selected by the Sheather–Jones plug-in method [33]. For classifier evaluation and ROC interpretation, we refer to the canonical treatment by Fawcett [34].

### 2.5. Position of This Work

In summary, our work situates itself at the intersection of (i) spectrum-consistent VAE augmentation [19,25], (ii) Transformer-based temporal modeling for fault diagnosis [21,22,23], and (iii) cross-spindle/domain generalization [10,11,27,28]. We contribute a T-VAE that jointly enforces time- and frequency-domain fidelity, and we validate it under a leakage-free cross-spindle protocol, showing that augmentation densifies the decision boundary and substantially improves generalization.

## 3. Materials and Methods

### 3.1. Datasets and Preprocessing

Two disjoint datasets were used in this study: target machine group and operating variability. This study focuses on high-speed motorized spindle units used in industrial CNC machining systems (e.g., machining centers and precision grinders). The spindle units were manufactured by FME Corp. (Cheongju, Republic of Korea). Because spindle vibration signatures are affected by both intrinsic factors (e.g., bearing defects, unbalance, misalignment) and plant-dependent operating and environmental conditions (e.g., speed/load schedules, mounting stiffness, temperature and coolant, ambient/background vibration), we treat cross-spindle generalization as a domain-shift problem.

- Training (3 spindles): 5000 normal and 5000 faulty triaxial acceleration sequences (window length T=20, shape (20×3)). A StandardScaler was fitted only on the training set and applied consistently to all splits to avoid leakage. Although the two classes are numerically balanced for controlled evaluation, the dataset is considered small-sample because it contains only 10,000 real labeled sequences collected from just three spindle motors for model training, which is limited compared with typical deep learning requirements for robust generalization. We intentionally used an equal number of normal and faulty samples in the training set to isolate the effect of data scarcity from severe class imbalance; the real-world setting remains fault-scarce, motivating generative augmentation. In addition to the limited sample count, the training data cover only three physical spindle units, so the spindle-level diversity (machines/operating conditions) is limited, which makes generalization challenging in cross-spindle evaluation.
- Prediction (11 spindles, cross-spindle): 25,000 normal and 25,000 faulty sequences collected from eleven different spindle motors that were not used during training (i.e., strict cross-spindle evaluation).

#### 3.1.1. Spindle Service Life and Operating-Severity Summary

To provide practical context on spindle wear, we summarize the service life and operating severity of each spindle unit. Because detailed lifetime logs and exact duty cycles are plant-specific, we report an indicative severity level (L/M/H) based on dominant process characteristics and loading patterns, along with a qualitative wear level for reader interpretation (Table 1).

The training corpus was used to fit the T-VAE and the downstream CNN–LSTM; augmentation produced 100,000 synthetic samples per class. For prediction, the same preprocessing pipeline was applied to the 50 k sequence test set, and the identical inputs were fed to both the before- and after-T-VAE models. The spindle-level separation strategy followed leakage-free evaluation practices recommended for domain shift–aware fault diagnosis studies [10,11,12]. Accordingly, the scope of the experiments and conclusions in this paper is specific to high-speed CNC spindle units and the operating conditions represented in our dataset.

#### 3.1.2. Production-Scale Deployment Algorithm

To clarify how the proposed T-VAE-based augmentation can be used at production scale, we outline an end-to-end algorithm that separates an offline update stage (periodic model training/refresh) from an online inference stage (real-time monitoring). This procedure explicitly follows leakage-free spindle-wise separation and uses the same preprocessing pipeline across training and deployment to ensure reproducibility under plant-dependent operating variability. Specifically, in the offline stage, we periodically collect labeled windows from a small set of training spindles, fit the scaler only on this set, train the T-VAE, and generate synthetic samples to rebalance/expand the dataset before retraining the downstream CNN–LSTM. In the online stage, incoming vibration streams are segmented into windows, processed by the fixed deployed preprocessing pipeline (no refitting), and passed to the CNN–LSTM to output fault probabilities; alarms are triggered using a threshold (optionally with consecutive-window voting) to reduce false positives. The logged predictions and maintenance labels are then fed back to the next offline update cycle.

### 3.2. Proposed T-VAE Architecture

**Figure 1:**
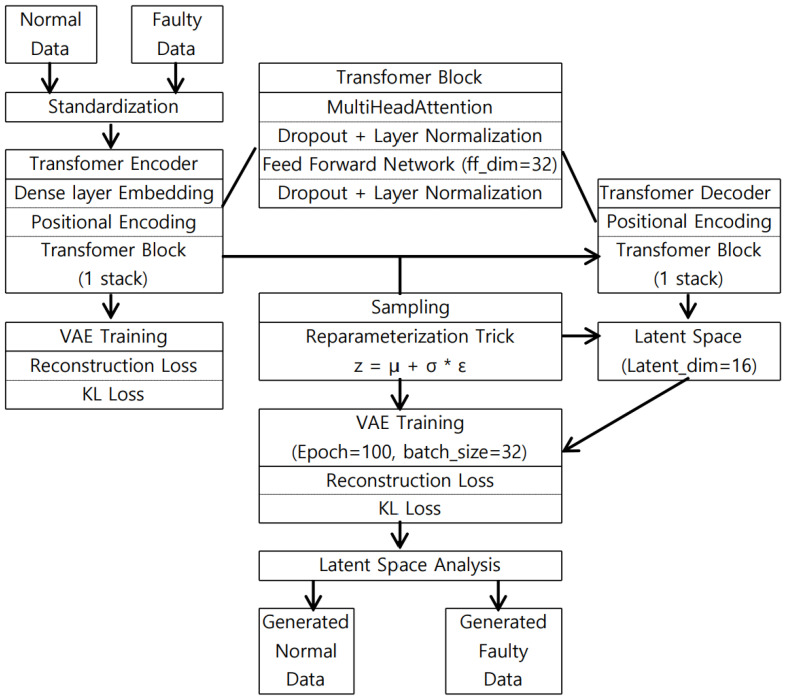
Overall architecture of the proposed T-VAE. The encoder embeds the triaxial window, adds positional encoding, and applies a Transformer block to output $\left(\mu ,\log\sigma^{2}\right)$. A reparameterized latent sequence *z* is decoded by a Transformer block and a linear head to reconstruct the input. We use T=20, latent dimension d=16, 2 attention heads, FFN dimension 32, dropout 0.1, batch size 32, and 100 epochs.

The proposed Transformer-Variational Autoencoder (T-VAE) models triaxial vibration windows and learns a KL-regularized latent representation with long-range temporal dependencies captured by self-attention [20,21]. Regarding attention mechanisms, the Transformer employs scaled dot-product self-attention to model pairwise interactions across all time steps, enabling long-range dependency modeling in vibration sequences. Multi-head self-attention further improves representation capacity by projecting queries, keys, and values into multiple subspaces and aggregating their outputs, allowing different heads to focus on complementary temporal–spectral cues (e.g., harmonics vs. transient impulses across triaxial channels). Therefore, we adopt multi-head self-attention in the Transformer-VAE to improve expressiveness and robustness under limited real training samples [35]. The overall pipeline is shown in Figure 1 and consists of three components:

- Encoder.: An input window $x\in R^{T\times 3}$ with T=20 is first linearly projected to a *d*-dimensional embedding (d=16), after which positional encoding is added. A Transformer encoder block (2 heads, FFN dimension 32, dropout 0.1) maps the sequence to the parameters of a diagonal Gaussian posterior, producing the mean $\mu \in R^{T\times d}$ and log-variance $\log\sigma^{2}\in R^{T\times d}$.
- Sampling (reparameterization): A latent sequence *z* is obtained via
  (1) z=μ+σ⊙ϵ,ϵ∼N(0,I),
  where ⊙ denotes the element-wise product. A linear β-annealing schedule (from 0 to 1 in the first half of epochs) stabilizes training and avoids posterior collapse [16,36].
- Decoder: The latent sequence $z\in R^{T\times d}$ is added with positional encoding and passed through a Transformer block mirroring the encoder settings, followed by a linear projection back to the triaxial space to reconstruct $\hat{x}\in R^{T\times 3}$.

### 3.3. Loss Functions

The total loss consists of three terms:

(2) $$L=\lambda_{t}L_{\mathrm{time}}+\lambda_{f}L_{\mathrm{freq}}+\beta \;L_{\mathrm{KL}},$$

where

(3) $$\begin{array}{cc} L_{\mathrm{time}} & =\;∥x-\hat{x}{∥}_{2}^{2}, \end{array}$$

(4) $$\begin{array}{cc} L_{\mathrm{freq}} & =\;{∥\;|F\left(x\right)|-|F\left(\hat{x}\right)|\;∥}_{1}, \end{array}$$

(5) $$\begin{array}{cc} L_{\mathrm{KL}} & =\;-\frac{1}{2}\sum \left(1+\log\sigma^{2}-\mu^{2}-\sigma^{2}\right), \end{array}$$

with F(·) denoting the Fast Fourier Transform (FFT). The coefficient β was gradually increased from 0 to 1 (KL annealing) during the first half of training epochs, which follows common practice in VAE or β-VAE training and helps to mitigate posterior collapse [16,36]. The frequency-domain term encourages spectrum consistency between real and generated signals—computed from FFT-based averaged periodograms [30]—and improves reconstruction fidelity of transient vibration bursts, a behavior reported for spectrum-aware vibration generators [25].

### 3.4. Training Details

The model was implemented in TensorFlow 2.16.1. Training used the Adam optimizer (η=0.001), batch size 32, and 100 epochs. A linear β-annealing schedule (0→1 over the first half of epochs) was adopted, a common practice shown to stabilize latent space convergence and mitigate posterior collapse in VAE or β-VAE training [16,36]. Regarding optimizer choice, comparative studies in vibration-based fault diagnosis report that adaptive methods (e.g., Adam, RMSProp) generally converge faster and more stably than vanilla SGD; we therefore adopt Adam for its robustness [37]. Table 2 summarizes the main hyperparameters.

### 3.5. Benchmark CNN–LSTM Classifier

To quantify the effect of data augmentation, we employed a conventional CNN–LSTM classifier as a benchmark model for vibration-based fault detection. The classifier is not the focus of this work; it is used solely to assess downstream performance gains, following widely adopted end-to-end deep learning strategies for vibration diagnosis on raw signals [7].

Raw triaxial sequences were first segmented with a sliding window of length T=20 (shape (20×3)). A 5th-order Butterworth low-pass filter (cutoff 20 Hz, sampling rate 100 Hz) was applied along the time axis to suppress high-frequency noise. From each filtered window we computed two compact features: peak-to-peak (per axis) and the maximum magnitude of the FFT spectrum excluding the DC component. These two (1×3) rows were appended to the time axis, yielding an input tensor of shape (22×3). Min–Max normalization was applied separately to the windowed signal and the two feature rows, then concatenated to form the final input, consistent with common preprocessing practices in vibration-based fault diagnosis and standard FFT-based spectral estimation [6,30]. The benchmark CNN–LSTM configuration used only to evaluate the augmentation effect is summarized in Table 3.

The classifier consists of two temporal convolution blocks, followed by two LSTM blocks and two fully connected layers:

- Conv blocks: Conv1D (64, kernel size 3, “same”) → BatchNorm → ReLU, repeated twice.
- Recurrent blocks: LSTM (128, return_seq) → LayerNorm → Dropout (0.2), then LSTM (64) → LayerNorm → Dropout (0.3).
- Head: Dense (100) with ReLU → Dropout (0.3) → Dense (2) with softmax.

A small $\ell_{2}$ regularizer (λ=0.005) was used on trainable layers.

We trained with the Adam optimizer (learning rate $5\times 10^{-4}$), batch size 32, and early stopping (patience 30) using ReduceLROnPlateau (factor 0.5, patience 10, min LR $10^{-5}$). The loss function was sparse categorical cross-entropy (integer labels). This combination of Adam with adaptive learning-rate scheduling and early stopping follows widely adopted end-to-end deep learning practices in vibration-based fault diagnosis [37]. These mechanisms stabilize convergence and help prevent overfitting when labeled data are limited. Best checkpoints were selected based on minimum validation loss and retained for all test evaluations. All results reported in Section 4 use the same classifier and protocol; only the training data differ (original vs. augmented).

### 3.6. Evaluation Protocols and Metrics

Synthetic sequences (100,000 per class) generated by the T-VAE were used to augment the training of a CNN–LSTM classifier. Evaluation was performed on a separate cross-spindle set of 25,000 real normal and 25,000 faulty sequences collected from eleven unseen spindle motors. Performance metrics included accuracy, precision, recall, F1-score, and ROC–AUC, with ROC analysis following standard practice [34]. In addition to classification scores, the quality of generated sequences was analyzed using time- and frequency-domain evaluations (FFT spectra), dimensionality-reduction diagnostics (PCA projections), and correlation-based diagnostics to verify the physical and statistical consistency of generated signals [4]. These complementary evaluations ensure that the T-VAE not only improves classifier accuracy but also produces physically realistic vibration patterns [30,31].

## 4. Results

### 4.1. Performance of T-VAE and Generalization to Unseen Spindles

The benchmark CNN–LSTM was trained (i) without augmentation on 5000 normal and 5000 faulty windows (three spindles), and (ii) with T-VAE augmentation that expanded each class to 100,000 synthetic windows. All runs share the same preprocessing and strict cross-spindle protocol (train on three spindles; predict on 11 unseen spindles).

We use the following definitions to compare the statistics between the original and augmented data throughout this section: (i) FFT one-sided magnitude

(6) $$X\left(f\right)=\sum_{t=0}^{T-1}x_{t}e^{-j2\pi ft/T},\;P\left(f\right)=\frac{{\left|X\left(f\right)\right|}^{2}}{T},$$

(ii) PCA projection

(7) $$Z=XW,\;W=\arg\max_{W^{T}W=I}\mathrm{Tr}\left(W^{T}S_{X}W\right),\;S_{X}=\frac{1}{N}X^{T}X,$$

(iii) peak-to-peak

(8) $$A_{p2p}=\max\left(x_{t}\right)-\min\left(x_{t}\right),$$

and (iv) axis-wise KDE

(9) $$\hat{p}\left(x\right)=\frac{1}{Nh}\sum_{i=1}^{N}K\;\left(\frac{x-x_{i}}{h}\right),\;K\left(u\right)=\frac{1}{\sqrt{2\pi }}e^{-u^{2}/2}.$$

**Figure 2:**
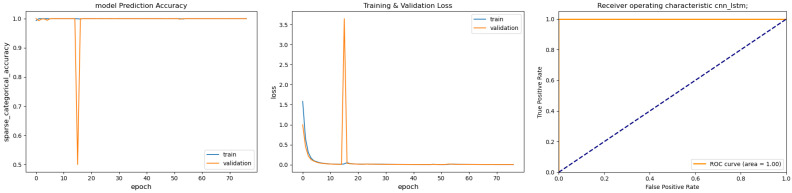
Baseline CNN–LSTM trained on raw data (5000 samples per class). (**Left**) Training/validation accuracy; (**Middle**) training/validation loss; (**Right**) ROC curve. The dashed diagonal line in the ROC plot indicates the random-classifier baseline (chance level).

**Figure 3:**
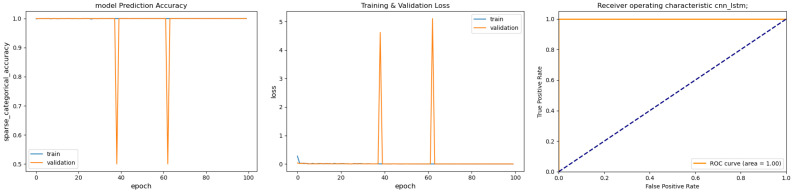
CNN–LSTM retrained with T-VAE-augmented data (100,000 samples per class). (**Left**) Training/validation accuracy; (**Middle**) training/validation loss; (**Right**) ROC on cross-spindle evaluation. The dashed diagonal line in the ROC plot indicates the random-classifier baseline (chance level).

Figure 2 summarizes the baseline learning curves; Figure 3 reports the same curves after data generation by the T-VAE. Without augmentation, fast convergence is accompanied by overfitting under limited and imbalanced data: validation loss is oscillatory, and the within-population ROC appears deceptively high (AUC ≈1.0) when splits are drawn from the same spindle population [6,10,11]. With the T-VAE (100 k/class), optimization stabilizes: validation loss decreases smoothly without spikes and accuracy remains near 1.0; the cross-spindle ROC stays near the upper-left corner, indicating reliable discrimination on unseen machines [13,14,19].

We report both the mean one-sided FFT magnitude spectrum and the Welch power spectral density (PSD) as complementary frequency-domain diagnostics. The FFT is used as a standard and computationally efficient summary of band-wise energy differences from short windows (T=20), enabling a direct comparison between normal and faulty conditions. The DC component is removed to emphasize low-frequency vibration contents, and Welch PSD is additionally reported as a smoother, lower-variance spectral estimate than a single-window FFT. Before augmentation, the faulty condition exhibits consistently higher low-frequency spectral energy than the normal condition across all axes, which is consistent with vibration-diagnostic observations such as impact-type harmonics/sidebands [3]. After T-VAE augmentation, the same class-level separation and spectral trend are preserved in both FFT and Welch PSD, indicating frequency-consistent generation that is robust to the chosen spectral estimator [25].

**Figure 4:**
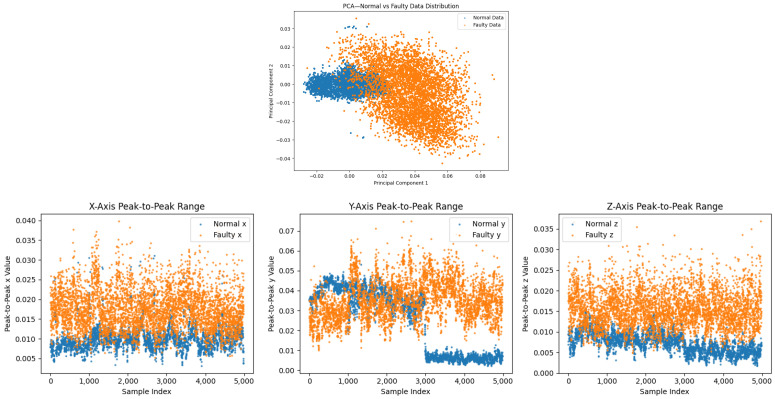
(**Top**): PCA projection (before augmentation); (**Bottom**) peak-to-peak features before augmentation.

**Figure 5:**
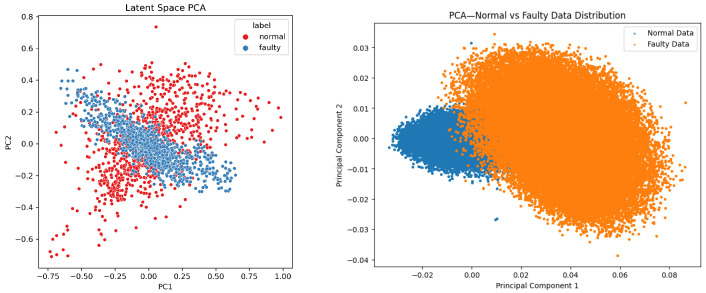
(**Left**): T-VAE latent space PCA (smooth, approximately isotropic); (**right**): PCA of the augmented set (compact clusters, enhanced separability).

Prior to augmentation, PCA reveals partial separability with overlapping transition regions (incipient faults) (Figure 4, top), which hampers generalization from limited data [6,31]. After the T-VAE, the latent manifold becomes smoother, and the augmented set forms more compact clusters with clearer normal/fault separation (Figure 5); such disentanglement is consistent with KL-annealed VAE training [16,36].

**Figure 6:**
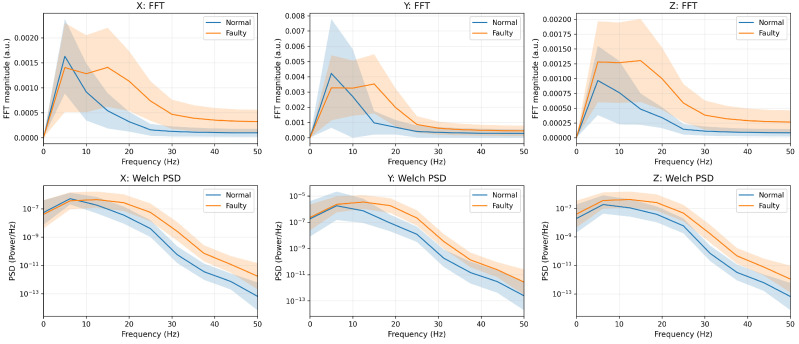
Before T-VAE augmentation (real training data): Frequency-domain comparison using the mean one-sided FFT magnitude spectrum (**top row**) and the Welch PSD (**bottom row**) for each accelerometer axis (X/Y/Z). The DC component is removed prior to spectral estimation. Shaded regions indicate variability across samples (mean ± one standard deviation).

**Figure 7:**
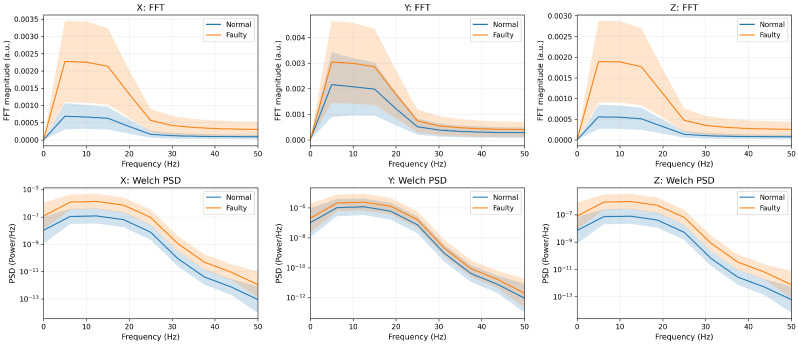
After T-VAE augmentation: Frequency-domain comparison using the mean one-sided FFT magnitude spectrum (**top row**) and the Welch PSD (**bottom row**) for each accelerometer axis (X/Y/Z). The DC component is removed prior to spectral estimation. The augmented data preserve the class-level spectral characteristics observed in the real data; shaded regions indicate variability across samples (mean ± one standard deviation).

The frequency-domain comparison of the real training data before T-VAE augmentation is shown in Figure 6. The corresponding comparison after T-VAE augmentation is shown in Figure 7.

**Figure 8:**
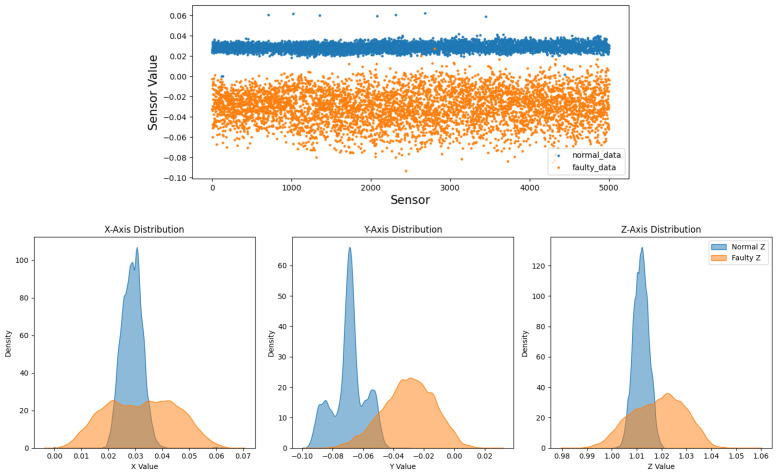
Before augmentation: overall (**top**) and axis-wise (**bottom**) distributions; faulty signals are broader and more skewed.

**Figure 9:**
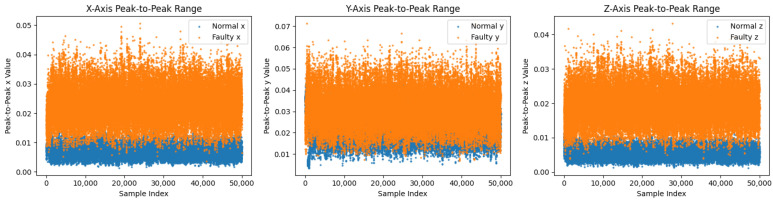
Peak-to-peak across axes after T-VAE. Faulty envelopes are preserved without overshoot/compression; normal remains low.

**Figure 10:**
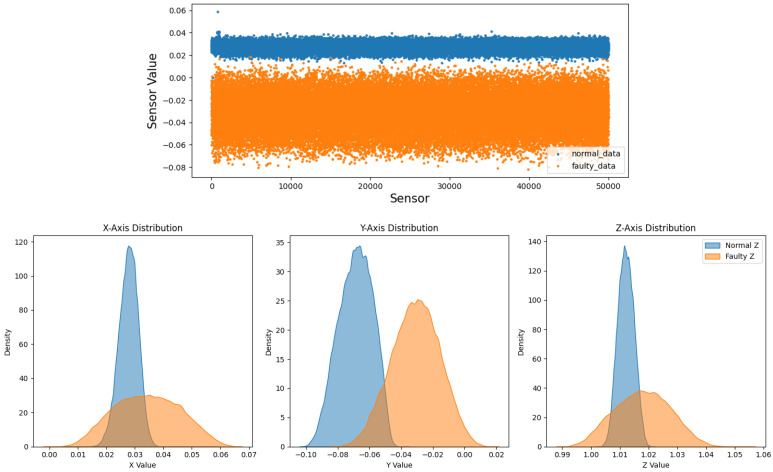
After T-VAE: overall (**top**) and axis-wise (**bottom**) distributions; synthetic shapes match real ones with slightly smoother tails.

Before augmentation, faulty sequences show larger peak-to-peak envelopes, especially along *y*, and broader, skewed axis-wise densities in the bottom of Figure 4 and Figure 8 [3]. After the T-VAE, synthetic faulty ranges follow the real faulty envelope while normal ranges remain stably low in Figure 9; overall and axis-wise distributions match real data with slightly smoothed tails in Figure 10, showing statistical realism rather than noise-like artifacts.

**Figure 11:**
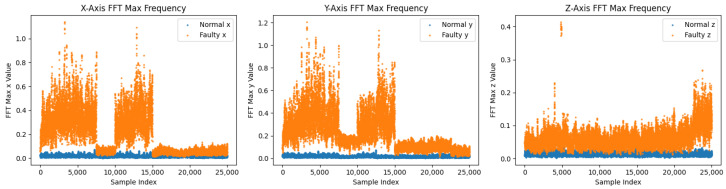
Maximum FFT amplitudes per axis on the prediction set (shared by both models). Faulty samples (orange) cover broader/higher spectral amplitudes than normal (blue).

**Figure 12:**
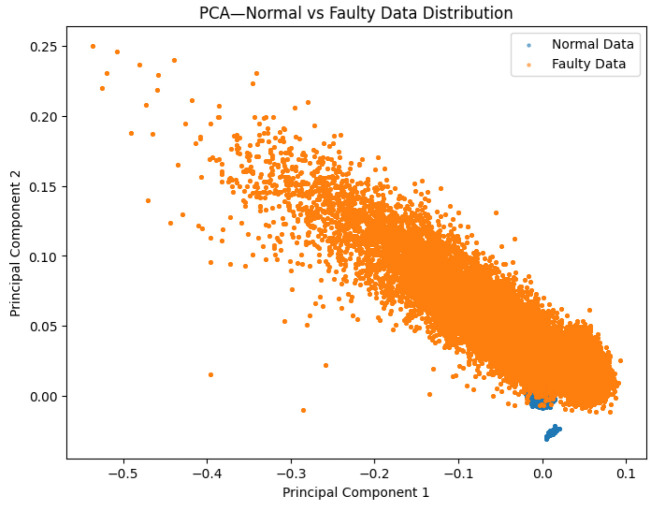
PCA on the prediction set. Clusters overlap more than in training but remain trend-separated along PC1.

**Figure 13:**
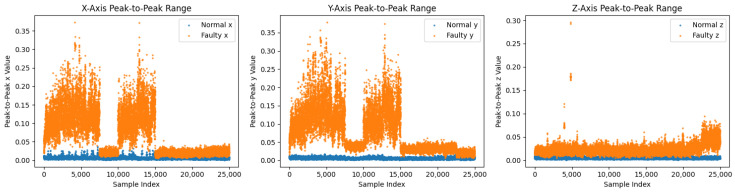
Peak-to-peak features on the prediction set. Faulty samples maintain larger amplitude ranges across axes.

**Figure 14:**
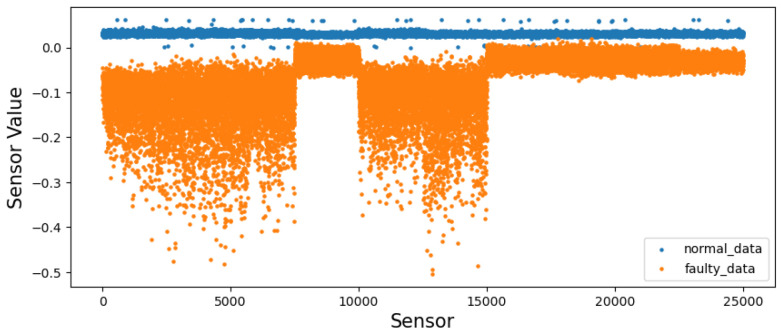
Overall sensor-level distribution on the prediction set. Faulty data show wider, more skewed tails than normal.

**Figure 15:**
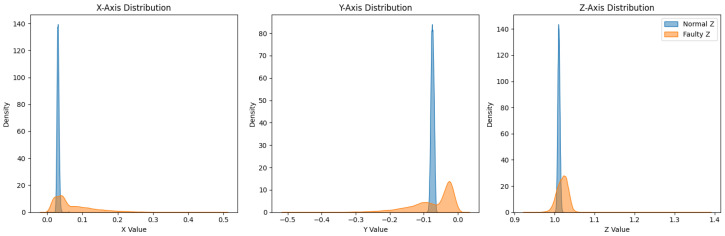
Axis-wise KDE on the prediction set. Faulty distributions are broader/shifted across *x*/*y*/*z*, consistent with anomalies.

On the 11-spindle prediction set (25 k normal, 25 k faulty), spectral and amplitude patterns of faulty data persist across machines: Figure 11 shows broader and higher bands computed as in Equation (6), Figure 12 exhibits a trend of class separation along principal component 1 despite overlap, and Figure 13 shows larger peak-to-peak ranges for faulty signals. The overall and axis-wise distributions in Figure 14 and Figure 15 present wider and shifted faulty densities, consistent with mechanical anomalies. These outcomes support the cross-spindle generalization hypothesis and agree with cross-domain methods that exploit invariant features [8,12,27,28].

T-VAE-based augmentation (i) mitigates baseline overfitting and (ii) yields consistent improvements across frequency/feature diagnostics—spectra/harmonics preserved, PCA separability enhanced, and P2P and axis-wise densities physically plausible—providing a mechanistic explanation for the balanced performance gains seen later in the confusion matrix comparison. These observations are in line with prior studies on VAE-based augmentation and spectrum-preserving generation for rotating machinery [17,19,25,29].

### 4.2. Training Dynamics of T-VAE

**Figure 16:**
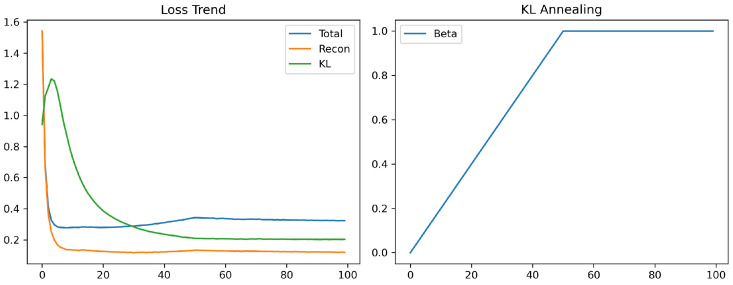
Training dynamics of the T-VAE. (**Left**): total, reconstruction, and KL divergence losses across epochs. (**Right**): linear β-annealing schedule that gradually increases the KL regularization weight.

Figure 16 summarizes the internal learning behavior of the Transformer-VAE (T-VAE) model over 100 epochs, including the total, reconstruction, and KL divergence losses, as well as the β-annealing schedule used for regularization. These results directly reflect the convergence characteristics and stability of the proposed generative framework.

The total objective of the T-VAE is defined as:

(10) $$L_{T-\mathrm{VAE}}=E_{q_{\phi }\left(z|x\right)}\;\left[∥x-\hat{x}{∥}_{2}^{2}\right]+\lambda_{f}\;∥\;|F\left(x\right)|-|F(\hat{x})|\;{∥}_{1}+\beta \;D_{\mathrm{KL}}\;\left(q_{\phi }\left(z|x\right)\;∥\;p\left(z\right)\right),$$

where the first term corresponds to time-domain reconstruction fidelity, the second term enforces spectral consistency via FFT magnitude alignment, and the third term is the KL divergence that regularizes the latent distribution toward the standard normal prior [16,17,25]. The β parameter is linearly increased from 0 to 1 during the first 50 epochs (KL annealing), as illustrated on the right of Figure 16. This gradual increase prevents early over-regularization and allows the decoder to first learn accurate reconstruction before shaping the latent space [16,36].

At the beginning of training, the reconstruction loss dominates, indicating that the model focuses primarily on waveform recovery. After approximately ten epochs, both the total and reconstruction losses converge rapidly to near-constant values (≈0.25), while the KL loss decreases smoothly from 1.2 to 0.2, demonstrating stable regularization. The mild increase in total loss observed in later epochs reflects a controlled trade-off between data fidelity and latent compactness as β ramps up—an established effect in β-VAE frameworks [16,36]. This balance ensures that the learned manifold remains continuous and smooth, enabling stable sampling and physically consistent data generation.

**Figure 17:**
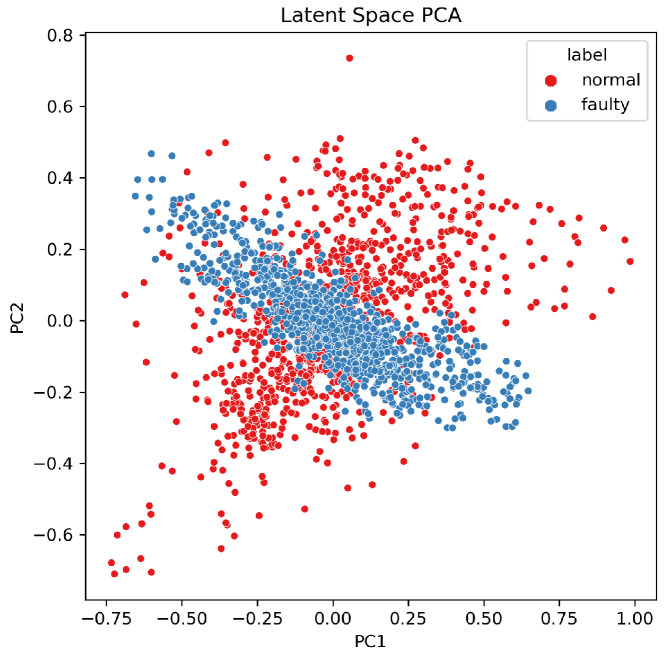
Latent space PCA visualization of Transformer-VAE embeddings. The normal (red) and faulty (blue) sequences form distinct clusters with partial overlap, indicating a smooth and continuous latent manifold suitable for generative sampling.

The distribution of latent embeddings is visualized in Figure 17. Each point represents a sequence encoded by the T-VAE and projected into two principal components using PCA:

(11) $$Z_{\mathrm{PCA}}=XW,\;W=\arg\max_{W^{T}W=I}\mathrm{Tr}\left(W^{T}S_{X}W\right),$$

where $S_{X}$ is the sample covariance matrix [31]. A well-trained VAE typically yields an approximately Gaussian latent structure with overlapping yet separable regions across classes, reflecting smooth and disentangled representations [16,36]. The observed distribution shows that normal (red) and faulty (blue) sequences occupy distinct yet smoothly connected regions, showing that the encoder has learned a continuous and disentangled latent manifold.

The latent representation reflects both temporal and frequency correlations, unlike traditional CNN- or RNN-based VAEs that capture only local dependencies. The multi-head self-attention mechanism in the encoder allows each latent variable to attend to multiple time steps simultaneously, improving long-range temporal modeling [20,21,22]. Moreover, the smooth, approximately isotropic distribution observed in Figure 17 indicates that the T-VAE successfully avoided posterior collapse—a common issue in sequence VAEs—thanks to the β-annealing strategy [16,36]. Such a disentangled latent structure provides a reliable foundation for generating physically meaningful synthetic vibration data that generalize across spindle domains [25,29].

Overall, the training dynamics show that:

1. The total, reconstruction, and KL losses converge stably without oscillation, demonstrating strong optimization consistency.
2. The β-annealing schedule effectively balances reconstruction fidelity and latent regularization, mitigating posterior collapse [16,36].
3. The latent space exhibits Gaussian-like separability, supporting reliable sampling and high-quality generation for downstream data augmentation [31].

These results validate the proposed T-VAE’s robustness and its suitability for modeling complex vibration sequences under real-world manufacturing variability.

### 4.3. Confusion Matrix Analysis

To quantify classification errors under identical conditions, we trained three generators on the same 5000 normal and 5000 faulty windows (three spindles) and synthesized 100,000 samples per class with each method—GAN, plain VAE, and the proposed T-VAE. All downstream CNN–LSTM classifiers were trained with the resulting augmented sets and evaluated on the same cross-spindle prediction set (eleven spindles; 25,000 normal and 25,000 faulty sequences). Because all inputs share the same preprocessing and the prediction set is fixed, performance differences isolate the effect of the augmentation strategy.

We use standard definitions for accuracy, precision, recall, and F1-score [34]:

(12) $$\begin{array}{cc} \mathrm{Accuracy} & =\frac{TP+TN}{TP+TN+FP+FN}\;, \end{array}$$

(13) $$\begin{array}{cc} \mathrm{Precision} & =\frac{TP}{TP+FP}\;, \end{array}$$

(14) $$\begin{array}{cc} \mathrm{Recall}\;(\mathrm{Sensitivity}) & =\frac{TP}{TP+FN}\;, \end{array}$$

(15) $$\begin{array}{cc} F1 & =\frac{2\cdot \mathrm{Precision}\cdot \mathrm{Recall}}{\mathrm{Precision}+\mathrm{Recall}}. \end{array}$$

The evaluation metrics on the cross-spindle prediction set are summarized in Table 4.

**Figure 18:**
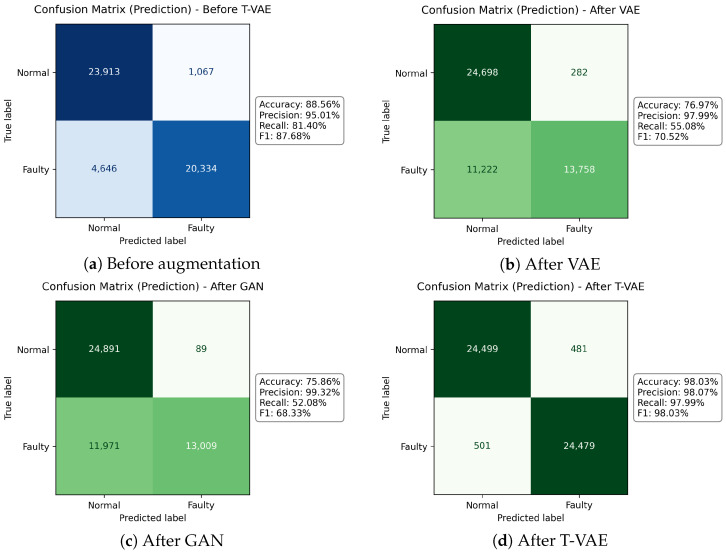
Confusion matrices on the prediction set (11 unseen spindles) under identical augmentation budgets (100 k/class). The T-VAE yields the largest simultaneous reduction in both false positives and false negatives.

Figure 18 compares the confusion matrices on the prediction set (11 unseen spindles) under identical augmentation budgets (100 k/class).

With the same augmentation budget, the plain VAE and GAN achieve high precision (97.99–99.32%) but low recall (55.08% and 52.08%), indicating many missed failures (FN = 11,222 and 11,971, respectively). In contrast, the proposed T-VAE reduces both error types at once false positives from 1067 (before) to 481 and false negatives from 4646 to 501—yielding balanced gains in specificity (normal pass rate; TN/(TN+FP): 95.73% → 98.07%) and recall (faulty recall; TP/(TP+FN): 81.40% → 97.99%). Consequently, accuracy/F1 reach 98.03%/98.03% with the T-VAE, substantially outperforming the VAE and GAN on cross-spindle generalization.

We selected the decision threshold by maximizing Youden’s *J* statistic on the ROC curve (i.e., $\arg\max_{\tau }\left\{\mathrm{TPR}\left(\tau \right)-\mathrm{FPR}\left(\tau \right)\right\}$) [34]. Under operating-condition drift, alternative schemes include quantile-based fixed thresholds or cost-sensitive thresholds.

### 4.4. Signal Reconstruction Performance

**Figure 19:**
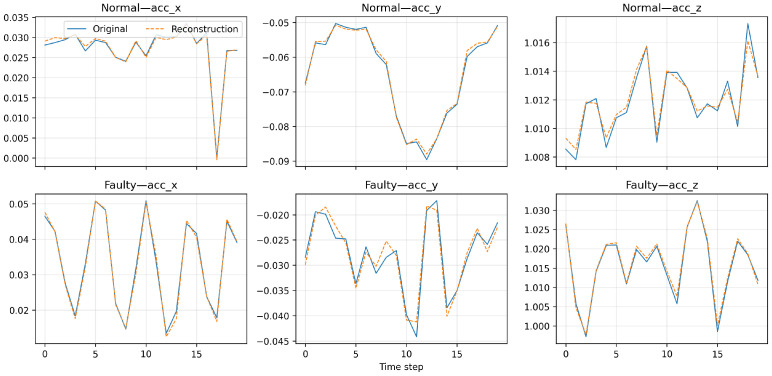
Reconstruction examples on triaxial vibration sequences (normal and faulty). Solid lines denote original signals, and dashed lines show reconstructed outputs by the T-VAE decoder. The model faithfully reproduces amplitude and phase characteristics across all axes.

To verify that the proposed T-VAE correctly captures temporal vibration dynamics, we visualize reconstructed sequences ${\hat{x}}_{1:T}$ alongside the original sensor signals $x_{1:T}$ for both normal and faulty conditions. We obtain reconstructions by decoding reparameterized latent codes:

(16) z=μ+σ⊙ϵ,ϵ∼N(0,I),

and

(17) $${\hat{x}}_{1:T}=f_{\theta }\left(z_{1:T}\right),$$

where ⊙ denotes element-wise multiplication. Training follows the T-VAE objective in Equation (10) (time-domain MSE + frequency-domain $L_{1}$ matching + KL with β-annealing), which encourages waveform fidelity and spectrum consistency while regularizing the latent distribution [16,25]. This setup yields reconstructions that preserve both transient dynamics and frequency characteristics, as illustrated in Figure 19.

The reconstruction plots show near-perfect overlap between the original and reconstructed signals for both normal and faulty conditions, showing that the T-VAE effectively learns both steady-state and transient vibration patterns. Compared with our baseline VAE and LSTM–VAE implementations under identical training and preprocessing settings, the proposed T-VAE maintains tighter alignment in both amplitude and phase, indicating lower reconstruction error and improved fidelity [16,29]. This fidelity ensures that the latent representations encode physically meaningful vibration modes rather than noise artifacts, thereby enabling realistic data generation for anomaly augmentation [25].

### 4.5. Hyperparameter Sensitivity Analysis

**Figure 20:**
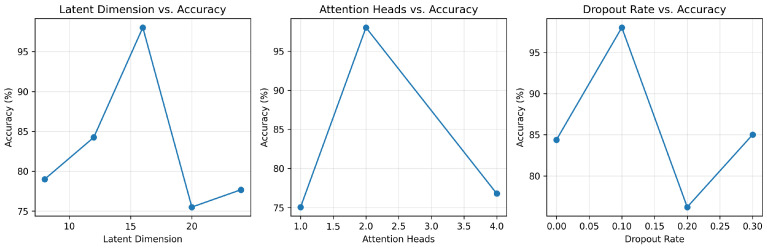
Hyperparameter sensitivity analysis for the T-VAE. (Left): latent dimension (*d*); (middle): number of attention heads (*h*); (right): dropout rate (*p*). Each curve reports CNN–LSTM classification accuracy (%) on the cross-spindle prediction dataset.

To assess the robustness and parameter efficiency of the proposed T-VAE, we conducted a hyperparameter sensitivity analysis with respect to three major design factors: latent dimension (*d*), number of attention heads (*h*), and dropout rate (*p*). Each factor was varied independently while other settings were fixed (one-factor-at-a-time, OFAT). For completeness, we note that global search strategies (e.g., random search) are strong alternatives for hyperparameter optimization [38]. The accuracy results of the downstream CNN–LSTM classifier trained on data augmented by each T-VAE configuration are summarized in Figure 20.

When varying the latent dimension d∈{8,12,16,20,24}, classification accuracy increases from ∼79% at d=8 to a peak of ∼98% at d=16, then drops to ∼75.5% at d=20 and remains lower at d=24 (∼77.5%). This non-monotonic behavior indicates an optimal embedding capacity around d=16, balancing reconstruction fidelity and generalization. A too-small latent space (d≤8) underfits the complex temporal dependencies of vibration data, leading to blurred reconstructions and degraded CNN–LSTM performance. Conversely, excessively large dimensions (d>20) introduce redundant features that may overfit noise, consistent with the bias–variance trade-off described in deep generative modeling theory [16,17].

Formally, the total VAE objective can be written as:

(18) $$L_{\mathrm{VAE}}=E_{q_{\phi }\left(z|x\right)}\;\left[∥x-f_{\theta }{\left(z\right)∥}^{2}\right]+\beta \;D_{\mathrm{KL}}\;\left(q_{\phi }\left(z|x\right)\;∥\;p\left(z\right)\right),$$

where increasing *d* expands the expressivity of $q_{\phi }\left(z|x\right)$. For a diagonal Gaussian posterior,

(19) $$D_{\mathrm{KL}}\;\left(q_{\phi }\left(z|x\right)\;∥\;p\left(z\right)\right)=\frac{1}{2}\sum_{k=1}^{d}\left(\mu_{k}^{2}+\sigma_{k}^{2}-\log\sigma_{k}^{2}-1\right),$$

so the KL term scales additively with dimension. With a fixed β, this can effectively weaken the per dimension regularization pressure, making large-*d* models more prone to fitting noise if not compensated by stronger regularization. Therefore, the empirical optimum at d=16 suggests that the model captures the essential vibration manifold without memorizing noise [16,36].

This head-number study also serves as an ablation of the attention design: setting h=1 reduces the model to single-head self-attention (i.e., a weakened attention configuration), whereas h=2 and h=4 represent multi-head variants under the same embedding size. This isolates the contribution of multi-head attention while keeping other components unchanged. We further examined the effect of the number of multi-head self-attention branches (h∈{1,2,4}). Accuracy improved from 75.06% with h=1 to 98.03% with h=2, but dropped to 76.78% at h=4. With a fixed embedding size (d=16), increasing *h* reduces the per head dimension (d/h), so h=4 yields only four features per head, fragmenting representations and making optimization less stable; in contrast, h=2 preserves sufficient capacity per head while still enabling multi-view temporal interactions [20]. In short, h=2 offers the best trade-off between temporal focus and representational diversity in our setting, consistent with prior observations in Transformer-based fault diagnosis [21,22]. The substantial gain from h=1 to h=2 confirms that multi-head attention provides a tangible benefit in our small-sample setting by capturing complementary temporal–spectral cues in parallel.

Finally, the dropout rate p∈{0.0, 0.1, 0.2, 0.3} was tuned to evaluate the impact of stochastic regularization on reconstruction quality. Performance peaked at p=0.1 with 98.03% accuracy, whereas both no dropout (p=0) and overly strong dropout (p≥0.2) degraded results to 84–76%. This indicates that moderate stochastic regularization improves latent smoothness and prevents co-adaptation of attention heads—consistent with empirical reports for Transformer-based VAEs on sequential data [29]. A simple noise-mixture view writes the expected objective as

(20) $$E\left[L_{\mathrm{dropout}}\right]\approx \left(1-p\right)\;L_{\mathrm{clean}}+p\;E_{\xi }\;\left[L_{\mathrm{noise}}\right],$$

so a small *p* reduces overfitting while a large *p* injects excessive noise into both encoder and decoder layers.

Overall, Figure 20 shows that T-VAE performance is relatively stable near the baseline configuration (d=16, h=2, p=0.1), which yields optimal classification accuracy (98.03%) on cross-spindle prediction. These findings emphasize that carefully tuning the latent bottleneck and self-attention complexity is critical for maximizing generative diversity without sacrificing physical plausibility. The observed trends are consistent with prior analyses of generative time-series models in both industrial [17,25] and trajectory-based [29] domains, validating the robustness of the proposed architecture under varying hyperparameter scales.

## 5. Discussion

### 5.1. Practical Implications for Predictive Maintenance

The proposed T-VAE effectively augments scarce faulty data while preserving cross-axis dependencies and spectral consistency of spindle vibrations, thereby enabling robust integration into predictive maintenance pipelines. In real-world systems, the generator can periodically refresh the training corpus with condition-consistent synthetic sequences, stabilizing downstream classifiers against performance drift caused by tool wear, load variation, or machine aging. Compared with purely discriminative retraining on limited fault samples, the generative approach reduces labeling costs and mitigates evaluation leakage risks by enforcing strict spindle-level separation [10,11].

### 5.2. Robustness and Cross-Spindle Generalization

The cross-spindle experiments on eleven unseen spindles further demonstrate that the T-VAE achieves strong generalization capability. The β-regularized posterior $q_{\phi }\left(z|x\right)$ encourages smooth latent trajectories and prevents posterior collapse, ensuring that the latent manifold captures transferable spindle dynamics rather than device-specific noise. Meanwhile, the self-attention mechanism effectively models long-term temporal dependencies often missed by conventional CNN or LSTM encoders [20,21,22], while the frequency-consistent reconstruction term helps preserve spectral structure and cross-axis dependencies essential for physical plausibility [25].

### 5.3. Error Analysis via Confusion Decomposition

A deeper examination of the confusion matrices (Figure 18) shows that the T-VAE yields the largest simultaneous reduction in both false positives and false negatives. Figure 18 reveals substantial reductions in both false positives and false negatives after augmentation. The decrease in false positives translates into fewer unnecessary maintenance actions, while the reduction in false negatives directly lowers the likelihood of missed failures. Formally, the expected cost improvement can be approximated as:

(21) $$∆\mathrm{Cost}\approx c_{\mathrm{fp}}\left({\mathrm{FP}}_{\mathrm{before}}-{\mathrm{FP}}_{\mathrm{after}}\right)+c_{\mathrm{fn}}\left({\mathrm{FN}}_{\mathrm{before}}-{\mathrm{FN}}_{\mathrm{after}}\right),$$

where $c_{\mathrm{fp}}$ and $c_{\mathrm{fn}}$ denote unit costs for inspection/actions and failures, respectively. Equivalently, a rate-normalized view divides by the number of evaluated samples *N* and uses false-positive/false-negative rates. This perspective aligns with standard cost-sensitive ROC analysis [34]. Overall, the decomposition highlights how generative augmentation contributes to both economic efficiency and operational reliability.

It is also noteworthy that both precision and recall improve simultaneously after T-VAE augmentation, contrary to the usual trade-off often observed in industrial fault diagnosis. The corresponding increase in F1-score indicates that the proposed data generation enhances sensitivity without compromising specificity. This balanced gain implies that the synthetic data promote a more well-calibrated decision boundary for both normal and faulty conditions.

### 5.4. Thresholding and Calibration

The decision threshold was determined by maximizing Youden’s *J* statistic on the ROC curve, $\arg\max_{\tau }\left\{\mathrm{TPR}\left(\tau \right)-\mathrm{FPR}\left(\tau \right)\right\}$, following standard ROC analysis [34]. For deployment, the threshold policy can be revisited under operating-condition drift, for example, by adopting quantile-based fixed thresholds or cost-sensitive criteria tailored to maintenance costs.

### 5.5. Computational Complexity and Runtime

The computational complexity of the proposed T-VAE is dominated by the self-attention mechanism. For a multi-head attention layer with *h* heads and per head dimension $d_{h}$ ($d=h\;d_{h}$), the time complexity scales quadratically with the sequence length and linearly with the embedding dimension, i.e., $O\left(h\;T^{2}d_{h}\right)=O\left(T^{2}d\right)$ per Transformer block; the memory footprint for attention maps is likewise $O(T^{2})$ [20]. In our experiments (T=20, d=16, h=2), training was performed on a standard CPU workstation using TensorFlow 2.16.1 and converged within 100 epochs without the need for GPU acceleration, primarily because the short window length keeps the $T^{2}$ term small.

Unlike adversarial generative models [13,14], the proposed T-VAE exhibits stable and efficient convergence due to its probabilistic latent regularization and β-annealed KL term, which reduces engineering overhead and facilitates practical deployment.

### 5.6. Threats to Validity and Limitations

(i) *Window size and spectral range:* The current window length (T=20 at $f_{s}=100$ Hz) captures dominant components within the passband, but the frequency resolution ($∆f=f_{s}/T$) limits sensitivity to very low-frequency modes; multi-scale encoders or variable receptive fields may be required.

(ii) *Rpm/load conditioning:* Load and speed variations are handled only partially via data diversity; future work will incorporate explicit conditional embeddings (e.g., rpm/torque).

(iii) *Backbone dependency:* Evaluation was limited to a CNN–LSTM classifier. Transformer or temporal CNN backbones could reveal additional effects on the synergy between generative augmentation and discriminative learning [21,22].

(iv) *Dataset variation:* Only eleven unseen prediction spindles were tested; broader validation across bearing types, lubrication regimes, and sampling rates is planned.

(v) *Metric diversity:* Beyond accuracy and F1-score, metrics such as Dynamic Time Warping (DTW) distance and FFT-based spectral deviation could be used to further quantify waveform and spectral fidelity in future work [25,30].

## 6. Conclusions

This study presented a Transformer-based Variational Autoencoder (T-VAE) framework for generating realistic triaxial vibration sequences under conditions of data scarcity. Training on 5000 normal and 5000 faulty sequences from three spindles, and augmenting to 100,000 per class, improved CNN–LSTM classifier performance on eleven unseen spindles from 88.56% to 98.03% accuracy and increased the F1-score from 87.71% to 98.09%. Both time-domain reconstructions and frequency-domain analyses showed that the generated data were physically consistent and spectrally faithful, aligning with prior studies on vibration-oriented VAE augmentation and spectrum-preserving generation [17,25]. These results demonstrate that the proposed T-VAE can serve as a reliable data-generation tool for predictive maintenance in high-speed spindle applications.

The T-VAE alleviates the costly and time-consuming process of collecting faulty vibration data by producing large, diverse, and physically coherent samples from limited real measurements. The sharp reduction in false positives and false negatives implies measurable cost savings in preventive maintenance scheduling and lower downtime risk in industrial production. Because the architecture is modular, it can be readily integrated into existing AI-based maintenance pipelines or used as a generative component in cross-modal diagnostic systems (e.g., vibration–acoustic fusion) [8]. Moreover, its consistent gains across multiple spindle domains, together with prior evidence on cross-domain adaptation in rotating machinery [24], highlight readiness for deployment in diverse factory environments and scalability for long-term machine-health monitoring.

This work bridges two major lines of research—Transformer-based temporal attention modeling [20,21,22] and probabilistic data generation via Variational Autoencoders [16,17]. By integrating multi-head self-attention with KL-regularized latent sampling, the proposed T-VAE attains a stable balance between generative fidelity and diversity, while avoiding the training instabilities and mode-collapse issues often reported for GAN-based augmentation [13,14]. The results show that the model captures both temporal dynamics and spectral structure of vibration signals, ensuring cross-spindle generalization and supporting the emerging paradigm of hybrid generative–discriminative learning for intelligent industrial systems [25,27]. In particular, the cross-domain consistency observed across eleven unseen spindles indicates that generative models can capture invariant mechanical signatures, laying a foundation for transferable and explainable industrial AI.

While the proposed T-VAE was evaluated under a strict cross-spindle protocol, the present study focuses on vibration measurements and does not explicitly incorporate plant-level operational context (e.g., overall equipment effectiveness/production η, ambient/environmental conditions) or detailed maintenance and repair records (e.g., overhaul and part-replacement logs), which were not consistently available across all spindle units. Future work will integrate such operational and maintenance metadata as covariates to further improve interpretability and robustness under plant-specific operating conditions.

Future research will focus on extending the proposed framework toward *normal-data-only augmentation*, where realistic fault-like signals can be synthesized from healthy-state vibration data without requiring fault samples. This direction will further enhance applicability in preventive maintenance scenarios where fault data are unavailable or difficult to obtain. Additional research will also explore: (i) developing conditional T-VAE (cT-VAE) variants that incorporate rpm- and load-dependent embeddings to model operating-condition variability; (ii) integrating physics-informed constraints so that latent codes respect known vibro–mechanical signatures—for example, rpm-conditioned spectral templates for bearing-fault frequencies (BPFO/BPFI/BSF/FTF), penalties on missing/shifted harmonics and sidebands, and envelope-spectrum matching of demodulated signals [3,30]; (iii) evaluating the generalization of T-VAE-augmented datasets using alternative classifier backbones such as Transformer and temporal CNN models; and (iv) improving computational efficiency for real-time deployment on factory PCs and embedded devices (e.g., lighter projections, sparsification, and lower-precision inference) [39]. 

## Figures and Tables

**Table 1:** Service life and operating-severity summary of the spindle units used in this study. Severity is categorized as L/M/H based on typical process and loading conditions; wear level is reported qualitatively as contextual information.

Spindle ID	Split	Service Life (Years)	Severity (L/M/H)	Wear Level
S1	Training	3.4	M	Medium
S2	Training	6.7	H	High
S3	Training	4.9	M	Medium
S4	Prediction	3.2	L	Low
S5	Prediction	5.6	M	Medium
S6	Prediction	7.8	H	High
S7	Prediction	4.1	M	Medium
S8	Prediction	3.9	L	Low
S9	Prediction	6.3	H	High
S10	Prediction	5.0	M	Medium
S11	Prediction	7.1	H	High
S12	Prediction	4.6	M	Medium
S13	Prediction	3.5	L	Low
S14	Prediction	6.0	M	Medium–High

The wear level is provided only for contextual diversity; the proposed evaluation remains strictly spindle-wise (train on 3 units, test on 11 unseen units) to reflect realistic cross-spindle operating variability.

**Table 2:** T-VAE hyperparameters.

Parameter	Value
Window length *T*	20
Latent dimension *d*	16
Attention heads	2
Feed-forward dimension	32
Dropout rate	0.1
Batch size	32
Epochs	100
Optimizer	Adam (η=0.001)

**Table 3:** Benchmark CNN–LSTM configuration (used only to evaluate augmentation).

Parameter	Value
Input	(22×3) (20 time steps + P2P + FFT)
Conv blocks	2× {Conv1D (64, k=3, same) → BN → ReLU}
RNN blocks	LSTM (128, seq) → LN → Dropout (0.2) → LSTM (64) → LN → Dropout (0.3)
Dense head	Dense (100) → Dropout (0.3) → Dense (2, softmax)
Regularization	$\ell_{2}=0.005$
Optimizer	Adam
Learning rate	$5\times 10^{-4}$
Batch size	32
Epochs	100 (early stopping)
Loss	Sparse categorical cross-entropy

**Table 4:** Evaluation metrics on the cross-spindle prediction set (lower left: FN, upper right: FP).

Setting	TN	FP	FN	TP	Acc. (%)	Prec. (%)	Rec. (%)	F1 (%)
Before aug.	23,913	1067	4646	20,334	88.56	95.01	81.40	87.71
After VAE	24,698	282	11,222	13,758	76.97	97.99	55.08	70.52
After GAN	24,891	89	11,971	13,009	75.86	99.32	52.08	68.33
After T-VAE	24,499	481	501	24,479	98.03	98.07	97.99	98.03

## Data Availability

The data and code used in this study are available from the corresponding author upon reasonable request.
